# Light squeezing enhancement by coupling nonlinear optical cavities

**DOI:** 10.1038/s41598-024-58447-3

**Published:** 2024-04-02

**Authors:** H. Jabri, H. Eleuch

**Affiliations:** 1 Higher Institute of Biotechnology of Beja, University of Jendouba, Beja, 9000 Tunisia; 2 Department of Applied Physics and Astronomy, University of Sharjah, Sharjah, 27272 United Arab Emirates; 3 College of Arts and Sciences, Abu Dhabi University, Abu Dhabi, 59911 United Arab Emirates; 4 Institute for Quantum Science and Engineering, Texas A&M University, College Station, TX 77843 USA

**Keywords:** Physics, Optical physics, Quantum physics

## Abstract

In this paper, we explore the squeezing effect generated by two coupled optical cavities. Each cavity contains a second-order nonlinear material and coherently pumped by a laser. Our results show that light intensity is strongly improved due to the presence of the nonlinearities and mainly depends on the detunings between external laser frequencies and cavity modes. More interestingly, the proposed scheme could enhance light squeezing for moderate coupling between cavities : the squeezing generated by one cavity is enhanced by the other one. For resonant interaction, highest squeezing effect is obtained near resonance. When fields are non resonant, squeezing increases near resonance of the considered cavity, but decreases for large detunings relative to the second cavity. Further, when the dissipation rate of the second cavity is smaller than the first, the squeezing could be improved, attaining nearly the perfect squeezing. While the temperature elevation has a negative impact overall on the nonclassical light, squeezing shows an appreciable resistance against thermal baths for appropriate parameter sets.

## Introduction

Nonlinear interactions in optical cavities systems are at the origin of the appearance of intriguing phenomena and observations. Starting from optical bistability and multistability^1–3^, several nonclassical features of light have been investigated such as squeezing^4–6^, sub-Poissonian photon statistics^7,8^, antibunching^9–11^, entanglement^12,13^, and Bell nonlocality^14,15^. In quantum optics, one of the field quadratures of squeezed states has smaller fluctuations compared to coherent light or a vacuum. The squeezing property of light is an essential resource in various applications. Not only it is used to reduce the noise level in optical communication^16,17^ and for the detection of extremely weak gravitational waves^18,19^, but also in quantum limited displacement sensing^20^, quantum cryptography^21–23^ and quantum computing^24–26^. In quantum computing, the reduction of noise below the shot-noise level is used to avoid the loss of encoded information during quantum computation, which could lead to an accumulation of errors. Therefore, by reducing tiny quantum-level fluctuations, scientists have been experimenting with squeezed light to reduce information loss^24^. Furthermore, the most notable application of squeezed light is to increase the astrophysical limits of gravitational-wave detectors including the laser interferometer gravitational-wave observatory (LIGO)^27^ and the gravitational-wave observatory (GEO 600) detectors^28^.

Squeezing property of light has been widely investigated in optics. This includes various systems and platforms such as two-level atomic system^29–34^, optomechanical systems^35,36^, quantum well cavity^37–42^ and many others. In all cases, the existence of optical nonlinearities, of second or third degree, is needed for the occurrence of the effect. For example, this can be realized by inserting a second-order nonlinear material in a cavity, or by realizing in a quantum well the strong light-matter coupling regime with a high excitonic density. Then, the additional Kerr type nonlinearities that could appear by coupling quantum wells via electronic tunnelling is proved to improve the squeezing effect^40,42^.

In light of this, we propose here a scheme consisting of two coupled cavities containing $\chi ^{(2)}$ materials and we show how this association could enhance the squeezing produced by one cavity and we provide the fundamental system requirements for the effect occurrence. The paper is organized as follows. In section 2 we give the Hamiltonian describing the system and we derive the corresponding evolution equations. Section 3 is consecrated to the study of the intensity of light inside cavities. In section 4, we determine the noise spectrum of the output light and we examine the squeezing property as a function of the frequency detunings, the amplitudes of squeezed light and the coupling strength between the cavities for resonant and off-resonant interactions.

## Hamiltonian and equations of motion

**Figure 1:**
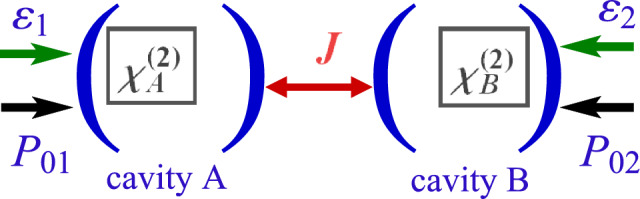
Two coupled cavities containing an optical parametric oscillator that generates a second-order optical nonlinearity. The resulting squeezed light from the nonlinear process in a cavity can be transferred to the other one. Cavity A (B) is pumped by a laser with an amplitude $P_{01}$ ($P_{02}$) with a decay rate $\kappa _{1}$ ($\kappa _{2}$).

The hybrid system under investigation, as shown in the schematic representation of Fig. 1, consists of two coupled cavities containing a $\chi ^{(2)}$ material. The cavity A has a frequency $\omega _{a}$ and a dissipation rate $\kappa _{1}$, while the frequency of cavity B is $\omega _{b}$ and its dissipation rate is $\kappa _{2}$. The pump field of amplitude $P_{01}$ ($P_{02}$) linearly drives cavity A (B). The pump field $\varepsilon _{1}$ drives the crystal of nonlinear susceptibility $\chi ^{(2)}_{A}$ placed in cavity A, while the crystal inside cavity B, of nonlinear susceptibility $\chi ^{(2)}_{B}$, is driven by the pump field $\varepsilon _{2}$. Directly pumping the nonlinear mediums results in a down-conversion process, which is responsible in the creation of highly correlated photon pairs and the appearance of squeezing. The interaction Hamiltonian of the whole system in the rotating wave approximation is given by

1 $$\begin{aligned} H=&-\Delta _{1} a_{1}^{\dagger } a_{1} -\Delta _{2} a_{2}^{\dagger } a_{2} +i J (a_{1}^{\dagger } a_{2} - a_{2}^{\dagger } a_{1}) \nonumber \\&+ i\frac{\varepsilon _{1}}{2} (a_{1}^{\dagger 2}-a_{1}^{2}) +i\frac{\varepsilon _{2}}{2} (a_{2}^{\dagger 2}-a_{2}^{2})\nonumber \\&+ i P_{01} (a_{1}^{\dagger } -a_{1})+ iP_{02} (a_{2}^{\dagger } -a_{2}) \end{aligned}$$

where $a_{1}^{\dagger }$ ($a_{2}^{\dagger }$) is creation operator of photons in cavity A (cavity B). $\varepsilon _{1}=\chi ^{(2)}_{A} \varepsilon _{pA}$ ($\varepsilon _{2}=\chi ^{(2)}_{B} \varepsilon _{pB}$) with $\varepsilon _{pA}$ ($\varepsilon _{pB}$) being the amplitude of the pump field that drives the crystal. The parameter *J* represents the strength of the photon hopping interaction between two cavities. $\Delta _{1}=\omega _{LA}-\omega _{a}$ ($\Delta _{2}=\omega _{LB}-\omega _{b}$) is frequency detuning between pump laser driving cavity A and cavity A mode (frequency detuning between pump laser driving cavity B and cavity B mode).

The dynamics of the system is governed by the master equation for the density matrix

2 $$\begin{aligned} \frac{d \rho }{dt} = -i \left[ H, \rho (t) \right] +{\mathscr {L}}\rho (t)^{loss} \end{aligned}$$

where ${\mathscr {L}}[q]=2 q \rho q^{\dagger } - \{q^{\dagger }q, \rho \}$ ($q \equiv a_{1}, a_{2}$) is the Lindblad superoperator for photonic dissipations in the two cavities. Then, the dissipative dynamics of the system is described by a set of quantum Langevin equations

3 $$\begin{aligned} \frac{da_{1}}{dt}&=\left( i\Delta _{1}-\frac{\kappa _{1}}{2}\right) a_{1}+J a_{2}+ \varepsilon _{1} a_{1}^{\dagger }+ P_{01}+\sqrt{\kappa _{1} }a_1^{in} \end{aligned}$$

4 $$\begin{aligned} \frac{da_{2}}{dt}&=\left( i\Delta _{2}-\frac{\kappa _{2}}{2} \right) a_{2}-J a_{1}+ \varepsilon _{2} a_{2}^{\dagger } + P_{02}+\sqrt{\kappa _{2} }a_2^{in} \end{aligned}$$

We assume that the cavity modes, relative to cavity A and B, are coupled to thermal reservoirs and the noise operators are $\delta$ correlated:

5 $$\begin{aligned} \langle a_{1}^{in}(t) a_{1}^{in\dagger }(t') \rangle&=(n_{a1}+1) \delta (t-t') \end{aligned}$$

6 $$\begin{aligned} \langle a_{1}^{in\dagger }(t) a_{1}^{in}(t') \rangle&=n_{a1} \delta (t-t') \end{aligned}$$

for cavity A, and

7 $$\begin{aligned} \langle a_{2}^{in}(t) a_{2}^{in\dagger }(t') \rangle&=(n_{a2}+1) \delta (t-t') \end{aligned}$$

8 $$\begin{aligned} \langle a_{2}^{in\dagger }(t)a_{2}^{in}(t') \rangle&=n_{a2} \delta (t-t') \end{aligned}$$

for cavity B, where $n_{a1}$ and $n_{a2}$ are the mean numbers of thermal photons in each cavity.

## Light intensity

Here, we study the intensity of light inside the cavities. However, as the difference between cavity A and cavity B is only the difference in system parameters, then by studying cavity A also implies studying cavity B. In the following, we consider cavity A which is applicable to cavity B. The evolution equations of the mean photon numbers are deduced from Eqs. (3) and (4) by removing the fluctuation terms

**Figure 2:**
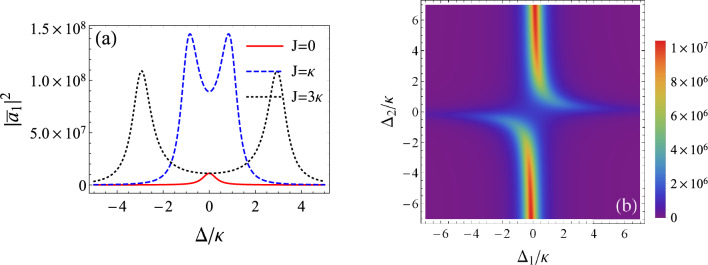
(**a**) mean photon number of cavity A as a function of the detuning $\Delta /\kappa$ for various couplings *J*. The parameters are $P_{01}=10^3\kappa$, $P_{02}=10^4\kappa$ and $\varepsilon _{1}=\varepsilon _{2}=0.2\kappa$. (**b**) density plot of the photon mean number versus the detunings $\Delta _{1}$ and $\Delta _{2}$ for $J=\kappa$, $P_{01}=P_{02}=10^3\kappa$, $\varepsilon _{1}=0.2\kappa$ and $\varepsilon _{2}=0.25\kappa$.

9 $$\begin{aligned} \frac{d {\bar{a}}_{1}}{dt}&=\left( i\Delta _{1}-\frac{\kappa _{1}}{2}\right) {\bar{a}}_{1}+J {\bar{a}}_{2}+ \varepsilon _{1} {\bar{a}}_{1}^{\dagger }+ P_{01} \end{aligned}$$

10 $$\begin{aligned} \frac{d {\bar{a}}_{2}}{dt}&=\left( i\Delta _{2}-\frac{\kappa _{2}}{2} \right) {\bar{a}}_{2}-J {\bar{a}}_{1}+ \varepsilon _{2} {\bar{a}}_{2}^{\dagger }+ P_{02} \end{aligned}$$

Eqs. (9) and (10) are similar with the temporal coupled-mode theory used in Refs.^43,44^. After solving these equations in the steady-state regime and considering the case of $\kappa _{1}=\kappa _{2}=\kappa$, we plot in Fig. 2a the light intensity of cavity A, $|{\bar{a}}_{1}|^{2}$, as a function of the detuning $\Delta /\kappa$ where we assume that $\Delta _{1}=\Delta _{2}=\Delta$. It can be seen that for $J=0$, meaning that cavity B is decoupled from cavity A, maximal intensity appears at resonance ($\Delta =0$) as a single peak, i.e., when the coherent driving field is at resonance with the cavity radiation frequency for cavity A. When cavity B is introduced with a photon interaction strength $J=\kappa$ and an amplitude of the pump field driving the crystal (which is directly linked to the degree of squeezing) $\varepsilon _{2}=0.2\kappa$, we observe that photonic intensity increases considerably and the spectrum has two peaks of same width centered around $\Delta =\pm J$. The distance separating the intensity peaks increases as the interaction is increased. This is noticed when $J=3\kappa$.

Now, we extend our study to non-resonant interactions ($\Delta _{1}\ne \Delta _{2}$). The density plot of Fig. 2b shows that the intensity has two branches of light. Away from this, the number of photons in the cavity is highly reduced. It is interesting to highlight that maximal intensity is obtained near resonance of cavity A, but away from resonance for cavity B.

Light intensity study is important in the measure that it provides the general conditions to obtain a non zero or maximal photon number in the cavity. This will guide us to find the system parameters for optimal degree of squeezing which will be discussed in the following section.

## Squeezing spectrum of the output light from cavity A

To determine the noise spectrum of the output field, it is more convenient to write the operators $a_{1}$ and $a_{2}$ that appear in Eqs. (3) and (4) as the sum of mean field values and fluctuations operators, as $a_{1}(t)={\bar{a}}_{1}+\delta a_{1}(t)$ and $a_{2}(t)={\bar{a}}_{2}+\delta a_{2}(t)$. The fluctuation parts $\delta a_{1}$ and $\delta a_{2}$ are supposed to be very small compared to the mean values ${\bar{a}}_{1}$ and ${\bar{a}}_{2}$. Then, the fluctuations satisfy the following equations

11 $$\begin{aligned} \frac{d}{dt} \delta a_{1}&=\left( i\Delta _{1}-\frac{\kappa _{1}}{2}\right) \delta a_{1}+J \delta a_{2}+ \varepsilon _{1} \delta a_{1}^{\dagger }+\sqrt{\kappa _{1} }a_{1}^{in} \end{aligned}$$

12 $$\begin{aligned} \frac{d}{dt}\delta a_{2}&=\left( i\Delta _{2}-\frac{\kappa _{2}}{2} \right) \delta a_{2}-J \delta a_{1}+ \varepsilon _{2} \delta a_{2}^{\dagger } +\sqrt{\kappa _{2} }a_{2}^{in} \end{aligned}$$

The phenomenon of squeezing occurs when a field quadrature has a lower noise than coherent light. As we are interested in optical field statistics of cavity A, it can be defined, by the general relationship, for a quadrature $A_{\theta }=a_{1}^{\dagger }e^{i\theta }+a_{1} e^{-i\theta }$

13 $$\begin{aligned} S_{\theta }\left( \omega \right) =&\int _{-\infty }^{+\infty }\left\langle A_{\theta } \left( t \right) \, ;\, A_{\theta }\left( 0\right) \right\rangle \;e^{-i\omega t}\;dt \nonumber \\ =&\int _{-\infty }^{+\infty } C_{A_{\theta }A_{\theta }}\;e^{-i\omega t}\;dt \end{aligned}$$

where $\theta$ is the field phase angle and $C_{A_{\theta }A_{\theta }}$ is the covariance of the quadrature $A_{\theta }$ defined by

14 $$\begin{aligned} C_{A_{\theta }A_{\theta }}(t)&=\left\langle A_{\theta }(t)\, ;\, A_{\theta }(0) \right\rangle \nonumber \\&=\left\langle A_{\theta }(t) \, A_{\theta }(0)\right\rangle - \left\langle A_{\theta }(t)\right\rangle \, \left\langle A_{\theta }(0)\right\rangle \end{aligned}$$

By writing the quadrature operator $A_{\theta }$ as the sum of a mean value and a fluctuation term, $A_{\theta }= \bar{A_{\theta }} + \delta A_{\theta }$, the covariance function $C_{A_{\theta }A_{\theta }}$ is interpreted as the average of the product of the fluctuations $\delta A_{\theta }$ at two instants separated by a time lapse *t*: $C_{A_{\theta }A_{\theta }}=\left\langle \delta A_{\theta }(t)\, \delta A_{\theta }(0)\right\rangle$. Then, the noise spectrum $S_{\theta }\left( \omega \right)$ represents the Fourier transform of the covariance $C_{A_{\theta }A_{\theta }}$

15 $$\begin{aligned} S_{\theta }\left( \omega \right) =&\int _{-\infty }^{+\infty }\left\langle \delta A_{\theta }\left( t \right) \delta A_{\theta }\left( 0\right) \right\rangle \;e^{-i\omega t}\;dt \end{aligned}$$

where, here, $\delta A_{\theta }$ represents a quadrature of the field relative to the fluctuation operators defined by $\delta A_{\theta } =e^{-i\theta }\delta a_1 +e^{i\theta }\delta a_{1}^{\dag }$. Working in the frequency domain makes the coupled differential equations given by Eqs. (11) and (12) simpler. Additionally, experimentally, the electric field fluctuations are more convenient to measure in the frequency domain than in the time domain. Indeed, the squeezing spectra can be easily measured in the outgoing light using a radiofrequency spectrum analyser connected to photodetectors. These spectra are directly related to the solutions of the linearized equations in the frequency domain $\delta a_{1}^{out}(\omega )$ and $\delta a_{1}^{out\dagger }(\omega )$. In fact, experiments allow us to measure the fluctuations of the output electric field in a quadrature defined by an angle $\theta$ with respect to some phase reference: $\delta A_{\theta }^{out}\left( \omega \right) =e^{-i\theta }\delta a_{1}^{out}\left( \omega \right) +e^{i\theta }\delta a_{1}^{out\dag }\left( \omega \right)$, and the measured spectra are given by $\left\langle \delta A_{\theta }^{out}\left( \omega \right) \delta A_{\theta }^{out}\left( \omega \right) \right\rangle$^45,46^. Given this, the noise spectrum $S_{\theta }( \omega )$ is thus written as

16 $$\begin{aligned} S_{\theta }\left( \omega \right) =&1+2C_{a_{1}^{\dag }a_{1}}\left( \omega \right) +C_{a_{1}a_{1}}\left( \omega \right) e^{-2i\theta }\nonumber \\&+C_{a_{1}^{\dag }a_{1}^{\dag }}\left( \omega \right) e^{2i\theta } \end{aligned}$$

The correlation function $C_{a_{1}^{\dagger }a_{1}}(\omega )$ is defined by $\langle \delta a_{1}^{\dagger }(\omega )\delta a_{1}(\omega ')\rangle =2\pi \delta (\omega +\omega ')C_{a_{1}^{\dagger }a_{1}}(\omega )$. The other correlations are defined in a similar way.

The noise spectrum of vacuum or a coherent state is independent of frequency and quadrature angle $\theta$, it is always equal to 1 and this corresponds to the shot noise or the standard quantum noise, $(S_{\theta }( \omega ))_{shot}=1$. A field is said to be squeezed if one of its quadratures has fluctuations in which one of the frequency components has a noise lower than standard quantum noise, meaning if there exist $\theta$ and $\omega$ such as $S_{\theta }( \omega )<1$. The optimized noise spectrum corresponds to the value of $\theta$ which maximizes the squeezing. By solving the equation $dS_{\theta }(\omega )/d\theta =0$, the angle $\theta$ satisfies the relation $e^{2i\theta _{opt}}=\pm C_{a_{1}a_{1}}/|C_{a_{1}a_{1}}|$. Then, the optimized squeezing spectrum of the emergent light is given by^47^

17 $$\begin{aligned} S_{opt}( \omega ) =1+2\left[ C_{a_{1}^{\dag }a_{1}}( \omega )-| C_{a_{1}a_{1}}( \omega )|\right] . \end{aligned}$$

The set of equations (11), (12) can rewritten in simpler form as: ${\mathscr {A}}.{\mathscr {U}} ={\mathscr {N}}$, where

18 $$\begin{aligned} {\mathscr {A}}=\left( \begin{array}{cccc} \alpha _{-} &{} - J &{} - \varepsilon _1 &{} 0 \\ J &{} \beta _{-} &{} 0 &{} - \varepsilon _2 \\ -\varepsilon _1 &{} 0 &{}\alpha _{+} &{} -J \\ 0 &{} -\varepsilon _2 &{} J &{} \beta _{+} \\ \end{array} \right) \end{aligned}$$

with $\alpha _{\mp }=i \omega \mp i \Delta _1+\frac{\kappa _1}{2}$ and $\beta _{\mp }=i \omega \mp i \Delta _2+\frac{\kappa _2}{2}$, and

19 $$\begin{aligned} {\mathscr {U}}=\left( \begin{array}{cccccc} \delta a_{1}(\omega ) \\ \delta a_{2}(\omega ) \\ \delta a_{1}^{\dagger }(\omega ) \\ \delta a_{2}^{\dagger } (\omega ) \end{array} \right) ,\, {\mathscr {N}}=\left( \begin{array}{cccccc} \sqrt{\kappa _{1}} \delta a_1^{in} \\ \sqrt{\kappa _{2}} \delta a_2^{in} \\ \sqrt{\kappa _{1}} \delta a_1^{in\dagger } \\ \sqrt{\kappa _{2}} \delta a_2^{in\dagger } \end{array} \right) \end{aligned}$$

The general solution for the photonic field of cavity A is simply a linear combination of the fluctuations given by:

20 $$\begin{aligned} \delta a_{1}(\omega )=&\sqrt{\kappa _{1}} L_{1}(\omega ) a_1^{in}+\sqrt{\kappa _{2}} L_{2}(\omega )a_2^{in} \nonumber \\&+\sqrt{\kappa _{1}} L_{3}(\omega )a_1^{in\dagger } + \sqrt{\kappa _{2}} L_{4}(\omega ) a_2^{in\dagger } \end{aligned}$$

where the $L_{i}(\omega ) (i = 1,2,3,4)$ functions are the elements of the inverse of the matrix ${\mathscr {A}}$, and written as:

21 $$\begin{aligned} L_{1}(\omega )= & {} {\mathscr {D}}^{-1}\left[ \alpha _+ \beta _- \beta _+-\alpha _+ \varepsilon _2^2+\beta _- J^2\right] \end{aligned}$$

22 $$\begin{aligned} L_{2}(\omega )= & {} {\mathscr {D}}^{-1}\left[ J^3+\alpha _+ \beta _+ J+\varepsilon _1 \varepsilon _2 J\right] \end{aligned}$$

23 $$\begin{aligned} L_{3}(\omega )= & {} {\mathscr {D}}^{-1}\left[ \beta _- \beta _+ \varepsilon _1-\varepsilon _1 \varepsilon _2^2-\varepsilon _2 J^2\right] \end{aligned}$$

24 $$\begin{aligned} L_{4}(\omega )= & {} {\mathscr {D}}^{-1}\left[ \alpha _+ \varepsilon _2 J+\beta _- \varepsilon _1 J\right] \end{aligned}$$

and ${\mathscr {D}}=det({\mathscr {A}})$ is given by

25 $$\begin{aligned} {\mathscr {D}}=&J^4+\alpha _- \alpha _+ \beta _- \beta _+-\alpha _- \alpha _+ \varepsilon _2^2-\beta _- \beta _+ \varepsilon _1^2 \nonumber \\&+\varepsilon _1^2 \varepsilon _2^2 +\alpha _- \beta _- J^2+\alpha _+ \beta _+ J^2+2 \varepsilon _1 \varepsilon _2 J^2 \end{aligned}$$

Using Eq. (20) and its complex conjugate, and the input-output relations linking the intracavity and the extracavity fields ($\delta a^{out}_{1}=\sqrt{\kappa _{1}}\delta a-a^{in}$ and $\delta a^{\dagger out}_{1}=\sqrt{\kappa _{1}}\delta a^{\dagger }-a^{\dagger in}$), then the expressions of the extracavity correlation functions are given by

26 $$\begin{aligned} C_{a^{\dag }a}^{out}( \omega )=&\kappa _{1}\Big [\kappa _{1} n_{a1} |L_{1}(-\omega ) |^{2} +\kappa _{1} (n_{a1}+1) |L_{3}(-\omega ) |^{2}\nonumber \\&+\kappa _{2} n_{a2} |L_{2}(-\omega ) |^{2}+\kappa _{2} (n_{a2}+1) |L_{4}(-\omega ) |^{2}\Big ]\nonumber \\&-2 n_{a1} \kappa _{1} \text {Re}[L_{1}(-\omega )]+n_{a1} \end{aligned}$$

27 $$\begin{aligned} C_{aa}^{out}( \omega )=&\kappa _{1}\Big [\kappa _{1} (n_{a1}+1) L_{1}(\omega ) L_{3}(-\omega ) \nonumber \\&+ \kappa _{2} (n_{a2}+1) L_{2}(\omega ) L_{4}(-\omega ) \nonumber \\&+ \kappa _{1} n_{a1} L_{3}(\omega ) L_{1}(-\omega ) +\kappa _{2} n_{a2} L_{4}(\omega ) L_{2}(-\omega )\Big ] \nonumber \\&-\kappa _{1}n_{a1} L_{3}(\omega )-\kappa _{1}(n_{a1}+1)L_{3}(-\omega ) \end{aligned}$$

## Light squeezing enhancement

**Figure 3:**
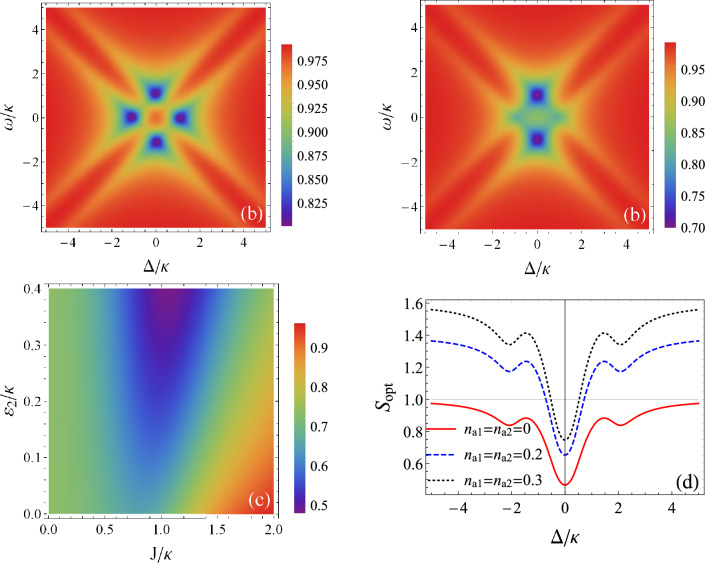
Noise spectrum of the output light as a function of the detuning $\Delta /\kappa$ and the frequency $\omega /\kappa$ for $J=\kappa$, $\varepsilon _{1}=0.1\kappa$ and $n_{a1}=n_{a2}=0$: (**a**) $\varepsilon _{2}=0$. (**b**) $\varepsilon _{2}=0.1\kappa$. (**c**) noise spectrum against the coupling $J/\kappa$ and the squeezed light amplitude of cavity B $\varepsilon _{2}/\kappa$ for $\Delta =0$, $\omega =\kappa$, $\varepsilon _{1}=0.2\kappa$ and $n_{a1}=n_{a2}=0$. (**d**) Noise spectrum against the detuning $\Delta /\kappa$ for various thermal photon mean numbers, for $J=\kappa$, $\omega =\kappa$ and $\varepsilon _{1}=\varepsilon _{2}=0.3\kappa$.

Note that the squeezing spectrum calculated above depends on the parametric down-conversion processes in cavity A and cavity B. These processes are manifested through parameters $\varepsilon _{1}$ and $\varepsilon _{2}$, respectively. In this section we analyze the squeezing properties of the emergent light from cavity A as a function of these nonlinearities as well as the mean photon numbers of thermal baths. First, we assume low temperature of the system, when phonon processes are suppressed, and we consider resonant case $\Delta _{1}=\Delta _{2}=\Delta$. The behavior of the squeezing spectrum is shown in Fig. 3a as a function of the detuning $\Delta /\kappa$ and the frequency $\omega /\kappa$. Parameters are such a that squeezing strength in cavity A is $\varepsilon _{1}=0.1\kappa$, photon hopping interaction strength is $J=\kappa$ and $\varepsilon _{2}=0$. We observe that emerging squeezing appears in four symmetrical peaks with respect to $\Delta =0$ and $\omega =0$. The nonclassical effect degree is of $20\%$. As we go away from these peaks, squeezing decreases and vanishes progressively when $S_{opt}(\omega )=1$, indicating a coherent light. Note here that cavity B contains a mixture of coherent, thermal and squeezed light. By choosing $\varepsilon _{2}=0$ and $n_{a}=n_{b}=0$, photons coming from cavity B are only coherent. Now, we take $\varepsilon _{2}=0.1\kappa$. As shown in Fig. 3(b), squeezing is enhanced and reaches $30\%$. The effect appears mainly in two peaks and remains localized around resonance.

**Figure 4:**
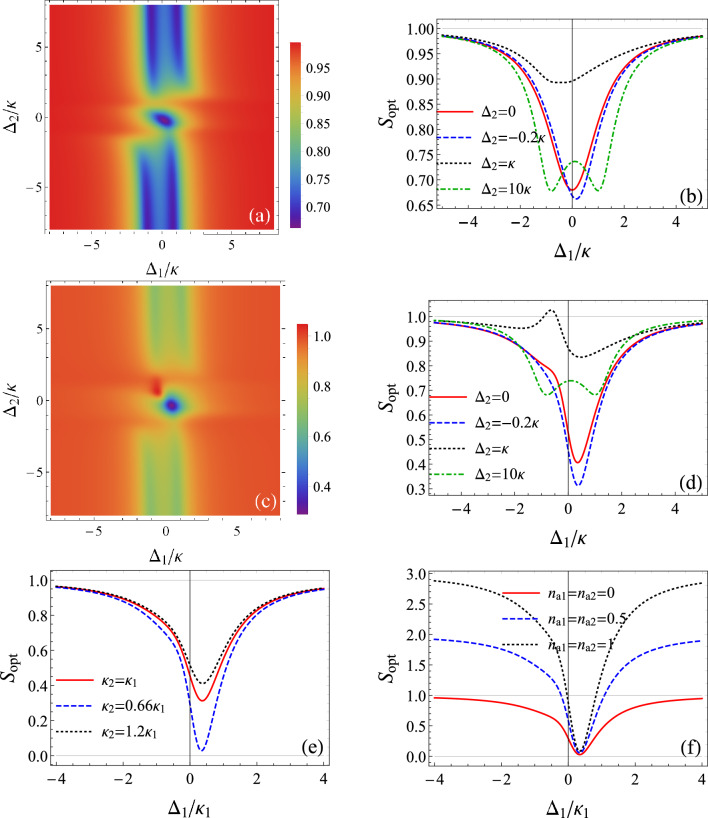
Noise spectrum of the output light as a function of the detunings $\Delta _{1}/\kappa$ and $\Delta _{2}/\kappa$ relative to cavity A and cavity B respectively for $J=\kappa$, $\varepsilon _{1}=0.2\kappa$, $\omega =\kappa$ and $n_{a1}=n_{a2}=0$: (**a**) and (**b**) $\varepsilon _{2}=0$. (**c**) and (**d**) $\varepsilon _{2}=0.25\kappa$. (**b**) and (**d**) are comparative plots for different detunings $\Delta _{2}$ corresponding to Figs. (**a**) and (**c**), respectively. (**e**) noise spectrum of the output light as a function of the detunings $\Delta _{1}/\kappa _{1}$ for $J=\kappa _{1}$, $\varepsilon _{1}=0.2\kappa _{1}$, $\varepsilon _{2}=0.25\kappa _{1}$, $\Delta _{2}=-0.2\kappa _{1}$, $\omega =\kappa _{1}$ and $n_{a1}=n_{a2}=0$. (**f**) noise spectrum of the output light as a function of the detunings $\Delta _{1}/\kappa _{1}$ for various thermal photon mean numbers and $\kappa _{2}=0.66\kappa _{1}$. The other parameters are the same as (**e**).

The effect of cavity B on the squeezed radiation of cavity A should be seen more closer. Indeed, Fig. 3c shows a density plot of the noise spectrum $S_{opt}(\omega )$ against the interaction parameter $J/\kappa$ and $\varepsilon _{2}$. It is clearly observed that the presence of cavity B strongly improves the squeezing generated by cavity A. The squeezing jumps from nearly $20\%$, when the cavities are decoupled ($J=0$), to more than $50\%$. This is possible for a coupling comparable to the cavity damping rate, $J\simeq \kappa$, and a squeezing strength from cavity B of $\varepsilon _{2}=0.4\kappa$. However, a stronger cavity couplings does not automatically generate higher squeezing. Thus, for $J=2\kappa$ for example, squeezing could disappear even in the presence of the two squeezed sources. Additionally, the coupling to the thermal baths has a negative effect on the squeezed radiation. Fig. 3d illustrates the noise spectrum versus $\Delta /\kappa$ for different thermal photon mean numbers. It can be seen that as the temperature increases, the nonclassical effect magnitude decreases and fluctuations appear above the shot noise level. Nevertheless, squeezing remains robust around resonance and shows a good resistance.

Now, we consider off-resonant interactions and we plot in Fig. 4 the noise spectrum against the detunings $\Delta _{1}/\kappa$ and $\Delta _{2}/\kappa$. If there is no squeezed photons coming from cavity B, the squeezing degree produced by cavity A is of $35\%$ when $\varepsilon _{1}=0.2\kappa$. Here, we can observe that there is a region around resonance corresponding to $\Delta _{2}\simeq \pm \kappa$ where squeezing could decrease. This behavior is attributed to the decrease of the light intensity in the cavity around these frequencies as shown in Fig. 2b. Except this, squeezing is obtained for wide ranges of the detuning $\Delta _{2}$. Then, $\Delta _{2}$ has a significant influence on the total squeezed radiation. Indeed, the increase of the detuning in cavity B could increase the squeezing in cavity A, as illustrated by the comparative plot of Fig. 4b. Then, the highest squeezing magnitude is obtained close to the resonance and also for large detunings relative to cavity B. This can be explained again based on the behavior of the light intensity which is important near resonance for cavity A and for high detunings for cavity B (Fig. 2b).

The situation changes by injecting squeezed photons from cavity B ($\varepsilon _{2}=0.25\kappa$). Indeed, we note a good enhancement of squeezing especially at resonance reaching more than $70\%$, meaning an increase of almost $35\%$ compared to the previous case (Fig. 4c). Here, highest squeezing is localized close to the resonance. Additionally, we notice that the choice of high detunings in cavity B will decrease the squeezing in cavity A (Fig. 4d).

Finally, we consider that the two cavities have different dissipation rates. The system parameters are now scaled to the damping rate of cavity A, $\kappa _{1}$. We observe that when $\kappa _{2}$ is greater than $\kappa _{1}$, the squeezing decreases compared to the case of identical cavities. However, if $\kappa _{2}$ is smaller than $\kappa _{1}$, the squeezing increases considerably and we could attain an amount of squeezing of $98\%$, approaching the perfect squeezing, when $\kappa _{2}=0.66\kappa _{1}$ (Fig. 4e). Indeed, when cavity B has smaller damping rate, the system is more decoupled from the environment. Then, the nonclassical effect will be more protected inside cavity B. In this case, the coupling between cavity A and cavity B enhances the squeezing of radiated light from cavity A. We also observe that the squeezing is unnoticeably affected by the temperature near the resonance for appropriate parameters sets (Fig. 4f).

## Conclusion

We investigated the squeezing of light produced by two coupled optical cavities containing second-order nonlinear crystals. We have shown that the coupling with a second cavity highly increases the photon intensity in the first cavity, and that light intensity is governed by the frequency detunings of both cavities. Indeed, to observe maximal light we should turn a cavity near resonance and the other away from resonance. We have shown also that the association of two nonlinear cavities could greatly enhance the squeezing compared to the single cavity case. The highest squeezing degree is obtained in a region approaching the resonance for both cavities. When the damping rate of the second cavity is smaller than the first, the squeezing is improved, attaining nearly the perfect squeezing.

## Data Availability

The datasets used and/or analysed during the current study available from the corresponding author on reasonable request. Results are generated using our analytical expressions in the manuscript with defined parameters.
